# Structural basis of lariat RNA recognition by the intron debranching enzyme Dbr1

**DOI:** 10.1093/nar/gku725

**Published:** 2014-08-14

**Authors:** Eric J. Montemayor, Adam Katolik, Nathaniel E. Clark, Alexander B. Taylor, Jonathan P. Schuermann, D. Joshua Combs, Richard Johnsson, Stephen P. Holloway, Scott W. Stevens, Masad J. Damha, P. John Hart

**Affiliations:** 1 Department of Biochemistry, The University of Texas Health Science Center, San Antonio, TX 78229, USA; 2 X-ray Crystallography Core Laboratory, The University of Texas Health Science Center, San Antonio, TX 78229, USA; 3 Department of Chemistry, McGill University, Montreal, Quebec H3A 0B8, Canada; 4 Northeastern Collaborative Access Team, Department of Chemistry and Chemical Biology, Cornell University, Ithaca, NY 14853, USA; 5 Program in Cellular and Molecular Biology, University of Texas at Austin, Austin, TX 78212, USA; 6 Department of Molecular Biosciences, The University of Texas at Austin, Austin, TX 78712, USA; 7 Institute for Cellular and Molecular Biology, University of Texas at Austin, Austin, TX 78712, USA; 8 Geriatric Research, Education, and Clinical Center, Department of Veterans Affairs, South Texas Veterans Health Care System, San Antonio, TX 78229, USA

## Abstract

The enzymatic processing of cellular RNA molecules requires selective recognition of unique chemical and topological features. The unusual 2′,5′-phosphodiester linkages in RNA lariats produced by the spliceosome must be hydrolyzed by the intron debranching enzyme (Dbr1) before they can be metabolized or processed into essential cellular factors, such as snoRNA and miRNA. Dbr1 is also involved in the propagation of retrotransposons and retroviruses, although the precise role played by the enzyme in these processes is poorly understood. Here, we report the first structures of Dbr1 alone and in complex with several synthetic RNA compounds that mimic the branchpoint in lariat RNA. The structures, together with functional data on Dbr1 variants, reveal the molecular basis for 2′,5′-phosphodiester recognition and explain why the enzyme lacks activity toward 3′,5′-phosphodiester linkages. The findings illuminate structure/function relationships in a unique enzyme that is central to eukaryotic RNA metabolism and set the stage for the rational design of inhibitors that may represent novel therapeutic agents to treat retroviral infections and neurodegenerative disease.

## INTRODUCTION

**Figure 1.**
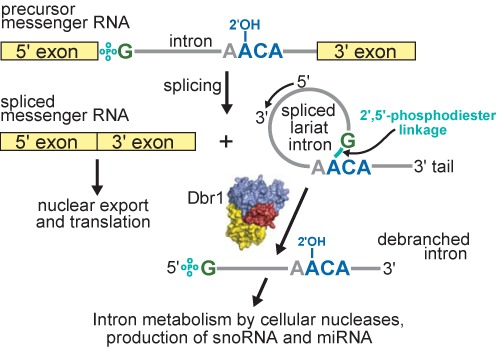
Overview of pre-mRNA splicing, lariat intron formation and subsequent debranching by Dbr1. The phosphate of the 2′,5′-phosphodiester linkage is teal. The coloring of branchpoint nucleotides is preserved throughout the manuscript. Domains in Dbr1 are colored as in Figure 2a.

The spliceosome excises introns from nascent messenger RNA (1) in the form of a lariat containing an unusual 2′,5′-phosphodiester linkage (2) (Figure 1). Hydrolysis of this linkage is rate-limiting in lariat degradation after splicing (3), and is required for efficient maturation of many small nucleolar RNAs (snoRNA) and micro RNAs (mirtrons) that are derived from intronic RNA (4,5). Supporting the critical role of Dbr1 activity in RNA metabolism, deletion of the *DBR1* gene in *Schizosaccharomyces pombe* causes severe growth and morphological defects (6), while deletion of the gene in higher eukaryotes is lethal (7), presumably due to their need of a larger complement of essential intronic snoRNAs and mirtrons.

The *DBR1* gene was first identified in a budding yeast genetic screen that sought to identify mutants defective in retrotransposition of the retrovirus-like Ty1 gene (3,8,9). Although the precise role of Dbr1 in retrotransposition is unclear, it has been suggested that a 2′,5′-phosphodiester bond might facilitate the strand transfer reaction preceding reverse transcription, and that hydrolysis of this bond is required to efficiently complete the process (10). This model is controversial (11,12) because direct evidence of an intermediate possessing a 2′,5′-phosphodiester bond is lacking. siRNA-mediated knockdown of Dbr1 expression has also been shown to reduce the efficiency with which the retrovirus HIV-1 can replicate (13). Retrotransposons and retroviruses are thought to share an ancestor due to their similar genetic structures and replication mechanisms (14) and, like the spliceosomal introns, likely evolved from an ancient group II intron (15). These genetic elements account for approximately one-third of the human genome (16) and are driving forces of evolution through the shuffling, replication and sharing of genetic material (17).

Knockdown of Dbr1 activity was recently demonstrated to suppress the toxic effects of the nucleic acid-binding protein TDP-43 in cell models of amyotropic lateral sclerosis (ALS) (18). TDP-43 binds to UG-rich regions of thousands of cellular RNAs (∼30% of the mouse transcriptome) (19)). The resulting elevated cellular pools of stable lariat RNA are thought to sequester pathogenic TDP-43, preventing it from aggregating and/or disrupting normal RNA metabolism (18). Small molecule inhibitors of Dbr1 may therefore be useful in the treatment of TDP-43 mediated ALS and the related neurodegenerative disease frontotemporal lobar degeneration (FTLD).

Sequence analyses predict Dbr1 consists of an N-terminal domain belonging to the metallophosphoesterase (MPE) superfamily of enzymes (20) and a C-terminal domain (CTD) lacking detectible sequence similarity to any other class of protein. MPE enzymes act upon a broad range of substrates, including 3′,5′-phosphodiester linkages (21), cyclic nucleotide monophosphates (22) and phosphorylated amino acids (23). Dbr1 is tuned to hydrolyze 2′,5′-phosphodiester RNA linkages in branched RNA and not the considerably more abundant canonical 3′,5′-phosphodiester linkages, although the molecular basis for this selectivity has remained elusive for several decades despite extensive biochemical characterization of the enzyme (24,25,26,27,28,29). To address this gap in knowledge, the 3D structure of the Dbr1 enzyme from the unicellular protist *Entamoeba histolytica* was determined using single crystal X-ray diffraction. The *E. histolytica* ortholog was used for this study due to its small size and ability to form diffraction-quality crystals. Numbering in the text refers to the *E. histolytica* enzyme. A sequence alignment of *E. histolytica* Dbr1 with other eukaryotic orthologs is presented in Supplementary Figure S1.

## MATERIALS AND METHODS

### Cloning, protein synthesis and purification

The codon optimized *DBR1* gene (GenScript) from *E. histolytica* was subcloned into a modified pET15b expression plasmid for heterologous expression as a C-terminal hexahistidine-tagged protein in *Escherichia coli*. Transformed cells (Rosetta pLysS, Novagen) were grown in 6 L of TB media with shaking at 37°C until the OD_600_ reached 0.5. The temperature was lowered to 16°C and the cells were shaken for an additional hour before isopropyl β-D-1-thiogalactopyranoside (IPTG) was added to a final concentration of 0.5 mM. The cells were incubated with shaking for an additional 20 h and harvested by centrifugation. Cell pellets were resuspended in immobilized metal affinity chromatography (IMAC) buffer containing 200 mM NaCl, 50 mM potassium phosphate pH 8.0, 50 mM imidazole pH 8.0, 10% glycerol, 1 mM TCEP supplemented with 2 mg of DNase I and lysed by sonication. Insoluble material was removed by centrifugation and the cleared supernatant was loaded onto a chelating sepharose column (GE Healthcare) pre-charged with Ni^2+^ and subsequently washed with 10 volumes of IMAC buffer. The protein was eluted in a single step using IMAC buffer containing 500 mM imidazole. Ethylenediaminetetraacetic acid was added to a final concentration of 100 mM to remove adventitiously bound metal ions. The protein was subsequently dialyzed into cation exchange buffer (100 mM NaCl, 20 mM HEPES pH 7.4, 10% glycerol and 1 mM TCEP) and purified on a HiTrapSP column. Purified Dbr1 was concentrated to 0.4–0.7 mM in cation exchange buffer. Inductively coupled plasma mass spectrometry revealed the purified protein retained substoichiometric quantities zinc ion. Prior to crystallization, MnSO_4_ or NiSO_4_ was added to the protein sample to a final concentration of 1 mM. The C14S variant of Dbr1 was purified using a similar protocol, except an N-terminal decahistidine-tagged protein was expressed in *Saccharomyces cerevisiae* under control of the SOD1 promoter. The cells were lysed by mechanical agitation with glass beads and the histidine tag was removed by digestion with tobacco etch virus protease prior to ion-exchange chromatography.

### Analytical ultracentrifugation

The oligomeric state of purified Dbr1 was analyzed by analytical ultracentrifugation sedimentation velocity in the UTHSCSA *Center for Analytical Ultracentrifugation of Macromolecular Assemblies* using a Beckman XL-I analytical ultracentrifuge equipped with absorbance optics and double-sector charcoal/Epon filled centerpieces. Dbr1 at a concentration of 0.3 mg/ml, corresponding to an absorbance of 0.5 at 280 nm, was centrifuged at 40 000 revolutions per minute at 20°C in buffer containing 20 mM HEPES pH 7.4, 100 mM NaCl. Velocity data were analyzed by the method of van Holde and Weischet (30) as implemented in the ULTRASCAN software package (31), which removes the contribution of diffusion to boundary spreading to yield the integral distribution of *s20,w* of all species in the sample. Consequently, a plot of boundary fraction versus *s20,w* will be vertical if the sample is homogeneous and will have a positive slope if the sample is heterogeneous. The molecular weight of Dbr1 in solution was calculated by 2D spectrum (32) and genetic algorithm (33) analyses as implemented in ULTRASCAN.

### Crystallization, data collection, structure determination and refinement

Crystallization screens were performed using a Phoenix crystallization robot (Art Robbins Instruments) with numerous commercial sparse matrix screens (Qiagen) in the UTHSCSA *X-ray Crystallography Core Laboratory*. After several hours of incubation in a sitting drop plate, clusters of needle-shaped crystals appeared in several conditions. The best crystals were grown at room temperature in hanging drops containing 1 μl of protein, 1 μl of water (or RNA at ∼1 mM) and 1 μl of a mother liquor containing 0.2 M Li_2_SO_4_, 0.1 M bis-tris pH 5.5, 25% PEG 3,350 and 8.5% glycerol. All specimens displayed epitaxial twinning to a degree that varied along the long axis of the needle-shaped crystals. ‘Brute force’ screening in combination with a microfocus X-ray source enabled the acquisition of diffraction data of quality sufficient for structure determination. All diffraction measurements were taken at the Advanced Photon Source, beamline 24-ID-C using an MD2 microdiffractometer and an ADSC Q315 area detector.

Diffraction data were integrated and scaled using HKL2000 (34) and XDS (35). The positions of 40 selenium sites (8 per protomer) were identified with SHELXD (36). Experimental phases were calculated using the multi-wavelength anomalous diffraction method (37) as implemented in the program SHARP (38). After density modification, the program RESOLVE (39) built over 80% of the protein structure into the experimental map automatically. Iterative cycles of manual building and refinement were performed using COOT (40) and PHENIX (41), respectively. All figures were generated with PyMOL (Schrödinger, LLC). Stereochemistry was analyzed with PROCHECK (42). Annealed omit maps were calculated in CNS (43,44).

### *In vivo* complementation assay

Wild-type and mutant Dbr1 constructs used for *in vivo* complementation analyses were prepared in bacterial expression vectors using conventional methods. Each variant was excised from its parent vector by restriction endonuclease digestion and ligated into vector pRS313-ySOD1 for recombinant expression under the control of the constitutive SOD1 promoter in *S. cerevisiae*. These plasmids were introduced into strain ySS4055 (MAT a *his3*, *leu2*, *lys2*, *trp1*, *ura3*, *dbr1::KAN*) by LiOAc transformation (45). Transformants were grown in synthetic defined medium without histidine (SD-HIS) to OD_600_ 1.0. Total RNA was prepared by the hot acid phenol method from these strains as well as the isogenic wild-type strain BY4741 prior to electrophoresis of 5 μg from each strain in a polyacrylamide gel (7% 19:1 polyacrylamide, 8 M urea, 1× TBE). RNA was transferred to nitrocellulose membranes and probed with ^32^P-labeled oligonucleotides complementary to the *ACT1* intron and to the U6 snRNA. Radioactivity was detected and quantitated by phosphorimaging.

Soluble proteins were extracted from the strains containing the indicated *E. histolytica* Dbr1 constructs by agitation with glass beads. Briefly, 3 ml of each strain were grown under selection in SD-HIS medium until OD_600_ of 1.5. Cells were harvested by centrifugation, washed in cold H_2_O and resuspended in three cell volumes of 20 mM Tris, pH 7.5, 150 mM NaCl, 8% glycerol, 10 mM β-mercaptoethanol and 1 μg/ml each of leupeptin and pepstatin. An equal volume of chilled glass beads was added. Eight cycles of 30 s vortexing, 1 min in ice water were performed. Insoluble material was removed by centrifugation and 15 μg soluble protein from each extract was electrophoresed through a lithium dodecyl sulfate (LDS) polyacrylamide gel electrophoresis gel (Invitrogen) and western blotted using an antibody against the HA tag common to all of the constructs.

### Branched RNA synthesis and *in vitro* debranching

RNA synthesis and debranching experiments were carried out as described elsewhere (46). Briefly, branched RNA compounds, AK51 and AK65, were synthesized on a glass solid support, utilizing branchpoint 2′-*O*-acetal-levulinyl adenosine synthons that permit stepwise installation of the 5′, 3′ or 2′-segments (24,47). Newly synthesized RNA molecules were purified by high performance liquid chromatography and size exclusion chromatography. The identities of the synthetic branched compounds were verified through mass spectrometry and debranching assays (46).

## RESULTS

### The overall structure of Dbr1

Dbr1 crystallized in space group *P*2_1_2_1_2_1_ with five molecules in the asymmetric unit. Diffraction data and protein structure refinement statistics are presented in Supplementary Table S1. Less than 1% of residues in each structure have torsion angles in the disallowed regions of a Ramachandran plot (48). The five protomers in the asymmetric unit are virtually identical, superimposing with an average root mean square deviation (RMSD) of 0.16 Å for backbone atoms. The observed packing interactions in the crystal are limited, consistent with analytical ultracentrifugation sedimentation velocity data indicating the protein exists as a ∼42 kDa monomer in solution (Supplementary Figure S2). The overall topology of Dbr1 is shown in Supplementary Figures S1 and S3.

**Figure 2.**
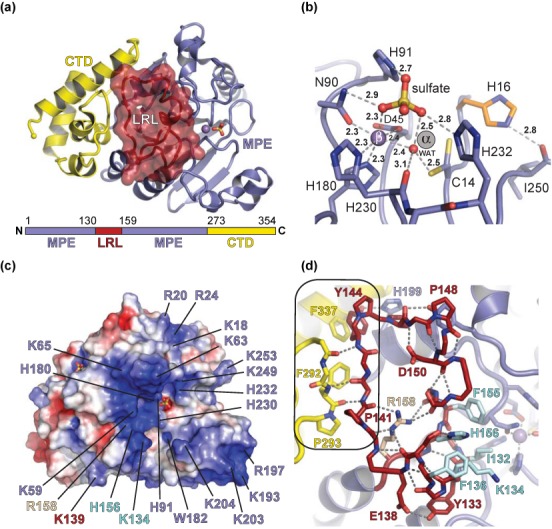
Structure of Dbr1 from *E. histolytica* highlighting structural elements involved in substrate recognition. (a) Dbr1 contains a MPE core domain (blue) with a 28 amino acid insertion termed the LRL (red surface, see text), and an 82 amino acid CTD (yellow). (b) The active site of Dbr1 in complex with a sulfate anion derived from the crystallization mixture shows a metal ion in the canonical MPE β pocket and a metal-free α pocket. The α pocket harbors a cysteine residue that is absolutely conserved in Dbr1 enzymes (Supplementary Figure S1) and adopts multiple conformers in the SO4•Dbr1 and 5GMP•Dbr1 co-structures presented here. (c) Surface electrostatic potential near the Dbr1 active site, contoured at ±4 *kT/e*. Positive potential is blue and negative potential is red. (d) The interface between the LRL and CTD (boxed) is stabilized by apolar and main chain-to-main chain hydrogen bonds. The conformation of the LRL is stabilized by an intricate hydrogen-bonding pattern that is centered on R158, which, is also invariant in Dbr1 enzymes (Supplementary Figure S1).

The N-terminal 261 residues adopt the MPE fold, characterized by adjacent α and β metal-binding pockets (Figure 2a and b). However, the α pockets in the four Dbr1 structures determined in this work are devoid of metal ions and an invariant cysteine residue (Cys14) in Dbr1 enzymes replaces the aspartic acid present in virtually all other members of the MPE superfamily (Figure 2b and Supplementary Figure S1). The Mn^2+^ ion in the β pocket is coordinated in an octahedral geometry by the side chains of Asp45, Asn90, His180, His230, a water molecule and a sulfate ion coming from the crystallization liquor (Figure 2b). As predicted in previous studies on the yeast enzyme (26), a conserved histidine residue (His91, Supplementary Figure S1) is poised to protonate the leaving group 2′O during hydrolysis of the 2′,5′-phosphodiester linkage in lariat RNA (Figure 2b). The electrostatic environment surrounding the active site is compatible with the binding of negatively charged RNA (Figure 2c).

Dbr1 possesses an internal loop element (residues 130–158) not found in other MPEs (Supplementary Figure S1). This loop, henceforth, referred to the lariat recognition loop (LRL), is positioned immediately adjacent to the active site and is stabilized by several interactions with the CTD as well as a network of intra-loop hydrogen bonds centered on an invariant arginine residue (R158, Figure 2d and Supplementary Figure S1). The CTD of Dbr1 (residues 262–354) consists of four alpha helices and three connecting loops, and is joined to the MPE domain by an extended linker region. The overall topology of the CTD is novel relative to other structures in the Protein Data Bank (PDB) (49), with a concave surface that ‘cups’ the MPE domain opposite the active site (Figure 2a).

### Structural elements critical for debranching activity

**Figure 3.**
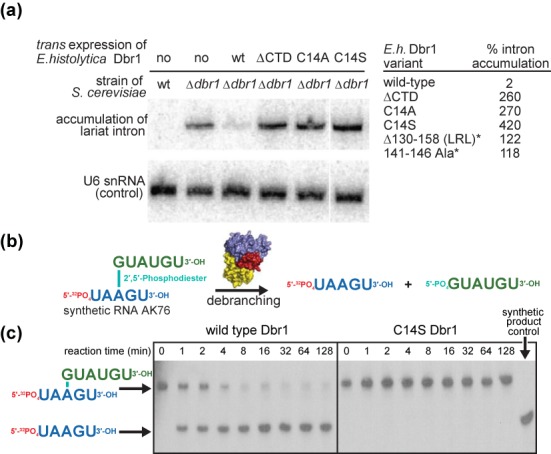
Activity of wild-type and mutant Dbr1 enzymes. (a) An *in vivo* complementation assay was used to assess debranching in *Δdbr1* stains of the model organism *S. cerevisiae*. Northern blotting allows monitoring of accumulated lariat RNA when Dbr1 has been deleted or inactivated. Levels of U6 snRNA were used to normalize each strain tested. (b and c) *In vitro* debranching assay using recombinant wild-type and C14S Dbr1 and a synthetic substrate analog (46).

We employed a series of activity assays to test hypotheses regarding the roles of the CTD, LRL and α pocket cysteine. An *in vivo* complementation assay was developed in a *Δdbr1* strain of *S. cerevisiae*, in which lariat RNA accumulates to high levels relative to wild-type *S. cerevisiae*. *Trans* expression of *E. histolytica* Dbr1 in this strain was sufficient to reverse the intron accumulation phenotype, confirming the enzyme is a *bona fide* lariat RNA debranching enzyme (Figure 3a). *Trans* expression of *E. histolytica* Dbr1 mutants lacking the CTD (residues 273–354), the LRL (residues 130–158, Supplementary Figure S4a), or the active site Cys14 residue (C14A or C14S) failed to reverse the intron accumulation phenotype (Figure 3a). A Dbr1 variant in which the LRL and CTD were retained but the interface between the two domains replaced by polyalanine (residues 141–146, Figure 2d and Supplementary Figure S1) was also incapable of reversing the intron accumulation phenotype, suggesting these elements act cooperatively in recognizing lariat RNA. Western blot analyses revealed all of these Dbr1 variants were expressed in the soluble fraction at levels similar to the wild-type enzyme (Supplementary Figure S4b).

The purified *E. histolytica* Dbr1 proteins used in the structural analyses were also tested in a gel-based assay for debranching activity. The wild-type enzyme was capable of debranching a model synthetic 11-nucleotide substrate containing a native branchpoint (46). However, the C14S mutant lacked detectible activity toward the same substrate (Figure 3b and c), demonstrating the active site cysteine residue invariant in Dbr1 enzymes is critical to the debranching reaction.

### Binding of substrate and product analogs

**Figure 4.**
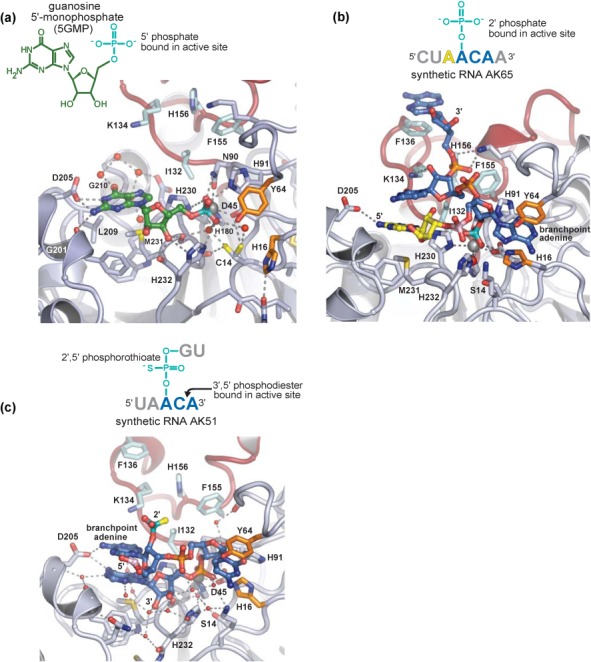
Structures of substrate and product analogs in complex with Dbr1. Water molecules are shown as red spheres, and mimics of the 2′,5′-phosphodiester moiety are colored teal. Remaining RNA and Dbr1 elements are colored as throughout the manuscript. (a) Guanosine 5′-monophosphate (5GMP) represents a product analog of the debranching reaction. In the 5GMP•Dbr1 co-structure, the 5′ phosphate is bound at the active site and contacts the β metal, N90, H91 and His232, while D205 makes sequence-specific contacts with the N1 and N5 atoms of the guanine base. (b) In the AK65•Dbr1(C14S) co-structure, a 2′ phosphate contacts the β metal, H16, N90, H91, H232 and a metal-associated water molecule that likely represents a catalytic nucleophile. The branchpoint adenine is sandwiched between H16 and Y64 (orange). There are extensive interactions between the 3′ tail of the lariat mimic and the LRL loop spanning amino acids 130–158. (c) Unexpected binding of a phosphorothioated RNA substrate analog. In two of the five chains in the crystallographic asymmetric unit, the phosphorothioate (teal) is not located in the active site. Instead, a 3′,5′-phosphodiester linakge contacts the β metal and N90, while the vicinal 2′ hydroxyl displaces His91 relative to the 5GMP•Dbr1 and Ak65•Dbr1 co-structures.

To visualize how lariat RNA is recognized by Dbr1, structures of the enzyme in complex with compounds that together mimic the branchpoint while remaining amenable to single crystal X-ray analyses were determined. Guanosine 5′-monophophate (5GMP) is essentially a 1 nucleotide ‘product’ of Dbr1-mediated lariat RNA debranching (Figures 1 and 4a). The structure of the 5GMP•Dbr1 complex reveals the 5′ phosphate of the product analog is in the same position as the sulfate in the ligand-free structure (Figure 2b). Despite the presence of 1 mM Mn^2+^ during crystallization, the α pocket in each of the four structures remains devoid of metal, suggesting that binding of manganese in the α pocket is not facilitated by binding of substrate or product in the Dbr1 active site. The guanine nucleotide of 5GMP makes direct or water-mediated hydrogen bonds with Gly201, Asp205 and Gly210 and apolar contacts with Leu209 and Met231 (Figure 4a). These interactions are consistent with the preference of Dbr1 for purines at the 2′ position relative to the lariat branchpoint (27), but would not necessarily prohibit binding of a pyrimidine.

The α pocket cysteine of Dbr1 was substituted to serine to prevent hydrolysis of larger RNA substrate analogs, such as AK65, a synthetic, linear RNA that possesses the 2′ phosphate at the conserved branchpoint adenosine but lacks the 2′ nucleotide that would be present in the complete lariat branchpoint. The structure of the AK65•Dbr1(C14S) complex shows that the enzyme engages the branchpoint nucleotide in a C2′-endo conformation that, in concert with stacking of the adenine base between His16 and Tyr64, places the pseudo-equatorial 2′ phosphate adjacent to the metal binding center and within hydrogen bonding distance of Asn90 and His91 (Figure 4b). There are few sequence-specific protein-RNA interactions at the branchpoint, consistent with the need for Dbr1 to hydrolyze lariat RNAs containing atypical branchpoint nucleotides (50). However, a pyrimidine is predicted to be suboptimal for aromatic stacking with His16 and Tyr64, consistent with the reduced activity observed toward cytidine branchpoints (27,51). Conversely, substitution by guanosine could promote tighter binding of the branchpoint nucleotide through hydrogen bonding interactions between N1 and N2 and the side chain of Gln47 (Supplementary Figure S5). The reduced activity toward branchpoint guanosine nucleotides (28) may therefore reflect product inhibition.

The structure also demonstrates that the LRL element unique to Dbr1 enzymes acts as a recognition module, with five of its residues (Ile132, Lys134, Phe136, Phe155 and His156) engaging the single-stranded arm 3′ to the branchpoint adenosine (Figure 4b). The interactions between RNA and the LRL are stabilized by secondary contacts between the LRL and the CTD involving residues 141–144 and 290–293, respectively (Figure 2d). In two of the five molecules of AK65 in the asymmetric unit, the adenosine 5′ to the branchpoint occupies a position similar to 5GMP in the 5GMP•Dbr1 complex. This binding mode is likely due to the preference for a purine at that site and by the absence of a 2′ nucleotide in AK65. In the other three AK65•Dbr1 complexes in the asymmetric unit, the 5′ nucleotide is disordered and not visible in the electron density maps. Thus, Dbr1 does not appear to recognize nucleotides 5′ to the branchpoint, but instead recognizes the branchpoint and flanking nucleotides directly and utilizes base stacking interactions between the 2′ and 3′ nucleotides to further stabilize the complex.

In an attempt to crystallize a branched RNA in complex with Dbr1, we initiated crystallization trials with the C14S mutant of Dbr1 and synthetic RNA AK51, a branched RNA containing a poorly-hydrolyzable phosphorothioate at the 2′ position (47) (Figure 4c). Ni^2+^ was used instead of Mn^2+^ because non-denaturing gel-shift experiments demonstrated the former conferred greater stability to the AK51•Dbr1(C14S) complex than did the latter (data not shown). In three of the five protomers in the asymmetric unit, the 2′,5′-phosphorothioate linkage was observed bound to the active site but only the branchpoint nucleotide was visible in the electron density. Surprisingly, the remaining two protomers in the asymmetric unit contained a 3′,5′-phosphodiester linkage in the active site (Supplementary Figure S6d). It is not immediately clear why two molecules of AK51 bound in this unexpected fashion although the substitution of the phosphorothioate or the presence of Ni^2+^ could conceivably play a role. Unlike 5GMP and AK65, which utilize numerous direct protein-RNA interactions, the binding of the 3′,5′ linkage in AK51 relies upon many water-mediated interactions and is therefore presumably weak.

## DISCUSSION

**Figure 5.**
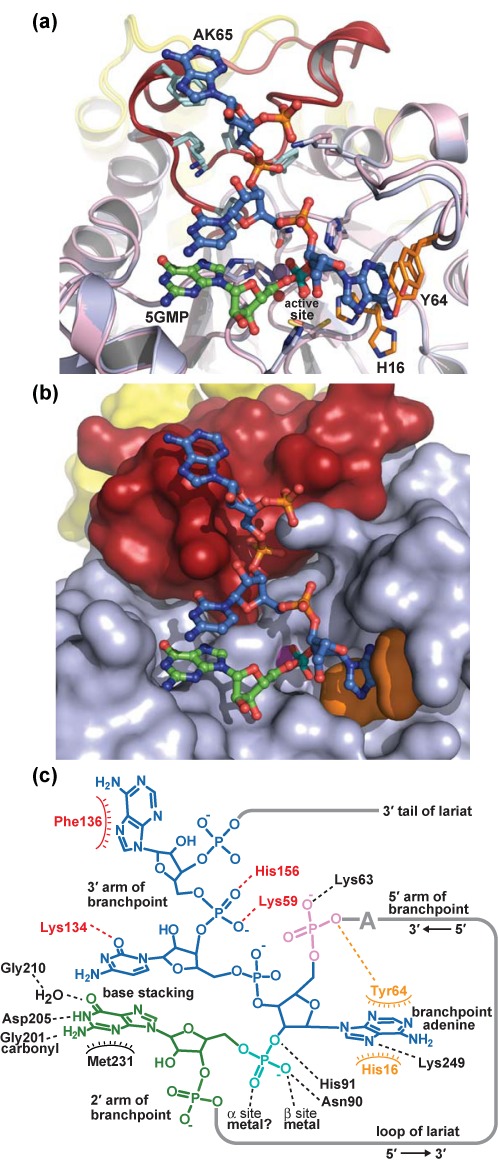
Model of lariat RNA bound to Dbr1. (a) Superposition of the entire 5GMP•Dbr1 co-structure (pink) with the entire AK65•Dbr1(C14S) structure (light blue). The 5′ adenosine in AK65 (yellow in Figure 4b) has been omitted for clarity. The LRL and CTD elements are colored red and yellow, respectively, in both structures. The RMSD of all protein atoms is 0.18 Å. His16 is displaced from the active site in the 5GMP•Dbr1 structure, but engages in direct stacking interactions with the branchpoint adenine base in the AK65•Dbr1(C14S) structure. (b) The superposition of these two structures produces a model of the lariat branchpoint bound to Dbr1 (shown in the same orientation as panel a). Protein domains are colored as in Figure 2a. (c) Schematic of contacts between Dbr1 and the lariat branchpoint. Dashed lines denote hydrogen bonding contacts.

When the protein components of the 5GMP•Dbr1 and AK65•Dbr1 complex structures are superimposed, the 5′-phosphate of 5GMP and the 2′-phosphate of AK65 also superimpose, providing a view of the intact lariat RNA branchpoint bound to the enzyme (Figure 5a and b). The 2′-nucleotide in the lariat branchpoint is derived from the 5GMP component, while the branchpoint nucleotide and flanking 5′ and 3′ nucleotides are derived from the AK65 component (Figures 1, 4a and b, 5b and 7b). In this model, base stacking between the 3′-cytidine and 2′-guanosine nucleotides exposes the branchpoint adenine base to be sandwiched between the side chain rings of Tyr64 and His16 (Figure 5b and c). The LRL makes multiple direct contacts with the 3′ tail of the lariat to further stabilize the conformation of RNA, while the CTD makes contacts with the LRL to help to stabilize the LRL–RNA interaction. Thus, it appears the CTD unique to Dbr1 enzymes plays an indirect but essential role in lariat RNA recognition. This role is corroborated by *in vivo* functional data, where targeted mutation of the LRL–CTD interface abrogates debranching activity (Figure 3a), although in the absence of direct binding data, the possibility that altering these elements might affect catalysis cannot be completely ruled out.

**Figure 6.**
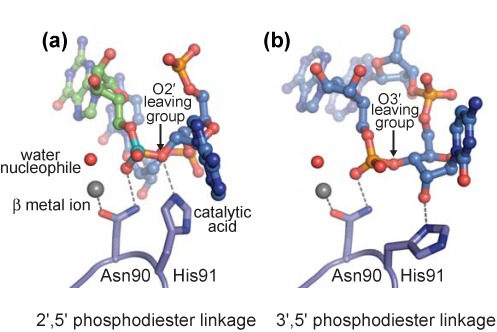
Structural basis for substrate discrimination. (a) In the model of lariat RNA bound to Dbr1, His91 is well-positioned to act as a catalytic acid and the metal-associated water molecule as the nucleophile in the hydrolysis of the 2′,5′-phosphodiester linkage. (b) In the AK51•Dbr1 co-structure, fortuitous binding of a 3′,5′-phosphodiester linkage reveals that the putative catalytic acid is displaced from the active site by the 2′ hydroxyl moiety, preventing hydrolysis of the RNA.

**Figure 7.**
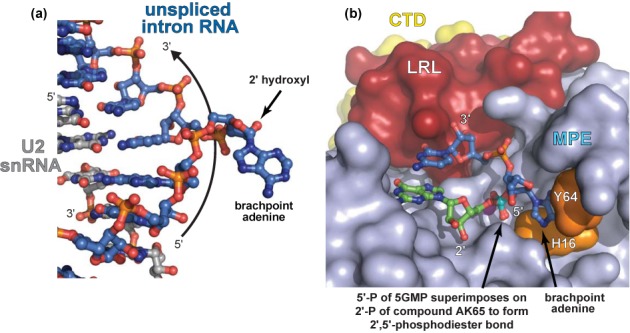
RNA base stacking interactions in Dbr1 and intronic RNA. (a) BPS intronic RNA (blue) in complex with a fragment of the U2 snRNA (gray) derived from PDB entry 3CGR (55). This structure, which provides a snapshot of intronic RNA immediately prior to formation of the lariat branchpoint, reveals that base stacking interactions between two nucleotides adjacent to the central adenosine serve to expose the branchpoint O2′ atom so that it is accessible for making the 2′,5′-phosphodiester linkage. (b) Model of lariat RNA in complex with Dbr1. Base stacking of adjacent nucleotides also exposes the central adenosine, suggesting a common structural mechanism for making and breaking 2′,5′-phosphodiester linkages.

There are few sequence-specific interactions between the model lariat and Dbr1, suggesting the conformation of the branchpoint is the major determinant for substrate recognition. This mode of recognition allows Dbr1 to hydrolyze lariat RNAs possessing atypical branchpoint sequences (BPSs) (27,50), a necessary consequence of Dbr1 being the principle pathway for hydrolyzing a diverse set of endogenous lariats within the cell. Notably, exogenous lariat introns, such as those generated from herpes simplex virus (52) and human papillomavirus (53), have a guanosine instead of adenosine at their branchpoints. Modeling a guanosine in the place of the branchpoint adenosine in the AK65•Dbr1 complex structure suggests it would be readily accommodated by the enzyme (Supplementary Figure S5).

The binding interactions between the composite lariat RNA branchpoint and Dbr1 suggest hydrolysis of the 2′,5′-phosphodiester linkage proceeds through a S_N_2 inversion mechanism in which bound metal acts as a Lewis acid in polarizing the non-bridging oxygen-phosphorous bonds to increase the electrophilic character of the phosphorous and simultaneously depress the pK_a_ of metal-bound water (Figure 2b) to increase its nucleophilicity. The structures suggest the putative trigonal bipyramidal transition state intermediate might be stabilized by interaction with the side chains of His16, Asn90 and His232 concomitant with proton transfer from His91 to the O2′ leaving group. It is unclear, however, whether the single metal ion observed in the Dbr1 active site represents the native, active form of the enzyme or an incomplete active site that binds a second metal ion concomitant with the branched RNA substrate (see Materials and Methods). The low barrier hydrogen bond between Cys14 and the active site water nucleophile (Figure 2b) could facilitate the action of Cys14 as a catalytic base in a mononuclear mechanism, as the O2′ leaving group is thought to have a lower pK_a_ than the O3′ leaving group (54). Additional studies are needed to fully elucidate Dbr1's catalytic mechanism.

Other determinants of substrate specificity are illuminated in the structure of the AK51•Dbr1 complex, in which two protomers contain a 3′,5′-phosphodiester linkage in the active site (Figure 4c). The binding of a 3′,5′-phosphodiester linkage requires rotation of the ‘branchpoint’ ribose, which induces a steric clash between the free 2′ hydroxyl and the side chain of His91, driving the imidazole moiety into a position that precludes it from acting as a catalytic acid (26) (Figure 6b). Binding of the 3′,5′-phosphodiester linkage also changes the trajectory of the 3′ strand of the RNA, which would likely prevent its association with the LRL in longer RNA molecules. Therefore, Dbr1 utilizes reduced catalytic potential in concert with lariat recognition to specifically hydrolyze the 2′,5′-phosphodiester linkage in lariat RNA.

The composite model of the lariat•Dbr1 complex bears a striking resemblance to unspliced BPS RNA when bound to a fragment of the U2 snRNA (55), despite the different internucleotide connectivity of these two RNAs. This might be expected as lariat debranching is in general terms analogous to a reversal of the first step of pre-mRNA splicing, and in both cases these conformations serve to expose the 2′O for either making or breaking a 2′,5′-phoshodiester linkage (Figure 7a and b). However, the conformation of our model lariat differs from that of a free lariat branchpoint analog in solution (56,57), where the branchpoint adenine of the analog makes a direct stacking interaction with the 2′ nucleotide. It is difficult to envision a way in which Dbr1 could engage this latter conformation of branchpoint RNA, thus underscoring the importance of the unique loop structures in Dbr1 for adapting the structure of lariat RNA to be compatible with the MPE catalytic machinery. These structural differences between free and bound lariat could have implications for the unusual stability of lariat RNA *in vivo* (3).

Although the CTD of Dbr1 lacks structural similarity (49) to any macromolecule in the PDB, there are multiple examples of structurally divergent domains at the C-termini of other MPE enzymes. The CTDs in other MPEs are typically located near (or are part of) the active site and/or can mediate assembly of higher order oligomers. However, the CTD of Dbr1 does not directly contact the active site and the enzyme is monomeric in solution (Supplementary Figures S2 and S8). In MPEs without debranching activity, regions analogous to the LRL element are smaller and adopt different conformations than in Dbr1. Thus, the unique CTD and LRL elements in *E. histolytica* Dbr1 appear to work in concert to facilitate lariat RNA recognition.

The structures suggest Dbr1 achieves its unique substrate specificity by providing a positively charged binding surface complementary in shape to that of the branchpoint and flanking nucleotides while simultaneously engaging in specific interactions with the extended 2′ and 3′ arms. The CTD, LRL and other amino acids that surround the active site, including K59, Y64, D205 and M231, constrain the suite of conformers available to bound RNA so that only the 2′,5′-phosphodiester linkage can be acted upon by Dbr1's catalytic machinery. Ancillary loop elements in other MPE enzymes play analogous roles, such as the CTD of Mre11 in constraining the orientation of double-stranded DNA when in proximity to the active site (58).

We anticipate these findings will help illuminate the relationship between Dbr1 activity and retroelement mobility by finally bridging the gap between structure and function in Dbr1. Mutation of canonical MPE active site residues has already been shown to inhibit retrotransposition, suggesting a critical role for debranching activity (8). It remains to be seen whether targeted mutation of elements that are unique to Dbr1 (Cys14, LRL and CTD) can also inhibit retrotransposition. Furthermore, it is not clear how mutation of putative a phosphorylation site located away from the active site (Tyr73, corresponding to residue Tyr68 in yeast) is able to suppress retrotransposition (8). This raises the possibility that Dbr1's role in retrotransposition may be under allosteric control *in vivo*, and such regulation may or may not extend to substrate binding and catalysis.

Understanding the precise mechanism of Dbr1 in retroelement mobility could serve as a gateway toward designing novel inhibitors of HIV1 replication (13). Similarly, small-molecule inhibitors of Dbr1 may also find utility in the treatment of neurodegenerative diseases, such as ALS and FTLD, where partial inhibition of Dbr1 allows accumulation of lariat RNAs to sequester toxic forms of the RNA-binding TDP-43 protein (18). Since complete inhibition of debranching activity is lethal (7), care must be taken to design inhibitors with very specific pharmacological properties. The structures presented in this work will help guide such efforts.

## ACCESSION NUMBERS

The atomic coordinates for the Dbr1•sulfate, Dbr1•5GMP, Dbr1•AK65 and Dbr1•AK51 structures have been deposited in the Protein Data Bank under accession codes 4PEF, 4PEG, 4PEH and 4PEI, respectively.

## SUPPLEMENTARY DATA

Supplementary Data are available at NAR Online.

SUPPLEMENTARY DATA
