# Supplementary material for: Structural basis of lariat RNA recognition by the intron debranching enzyme Dbr1
Source: Nucleic Acids Res. 2014 Aug 14;42(16):10845–55. doi: 10.1093/nar/gku725 (PMC4176325; doi:10.1093/nar/gku725)
Supplement: SUPPLEMENTARY DATA [file supp_42_16_10845__index.html]

Structural basis of lariat RNA recognition by the intron debranching enzyme Dbr1 — Structural basis of lariat RNA recognition by the intron debranching enzyme Dbr1 — SUPPLEMENTARY DATA 

# Structural basis of lariat RNA recognition by the intron debranching enzyme Dbr1

## SUPPLEMENTARY DATA

**Files in this Data Supplement:**

- SUPPLEMENTARY DATA
