# Supplementary material for: Structural basis of lariat RNA recognition by the intron debranching enzyme Dbr1
Source: Nucleic Acids Res. 2014 Aug 14;42(16):10845–55. doi: 10.1093/nar/gku725 (PMC4176325; doi:10.1093/nar/gku725)

SUPPLEMENTARY FIGURE LEGENDS, SUPPLEMENTARY FIGURES AND SUPPLEMENTARY TABLE

Supplementary Figure S1. Sequence alignment of Dbr1 enzymes from a diverse set of eukaryotic organisms. The MPE domain sequences are highly conserved across species. In contrast, the CTDs display greater phylogenetic variability. The secondary structural elements correspond to those observed in the structure of *E. histolytica* Dbr1, where the MPE domain is blue, the CTD is yellow, the LRL contacting both RNA and the CTD is red. Asterisks indicate invariant residues, two dots indicate high similarity and one dot indicates reduced similarity in alignment. α and β metal-binding pocket residues are highlighted in yellow, the aspartate that bridges the α and β pockets is black, RNA-binding residues located outside the metal-binding center are cyan, residues that stack with the branchpoint adenine are orange, and the invariant Arg residue of the LRL is tan. Boxed residues 141-146 were converted to alanine in the mutant with a disrupted LRL/CTD interface (see **Fig. 3a**).

Supplementary Figure S2. van Holde & Weischet combined distribution plot for purified *Entamoeba histolytica* Dbr1. The measured sedimentation coefficient of 3.4 S corresponds to a calculated molecular mass of ~42 kDa, which corresponds to the ~42 kDa mass of a Dbr1 monomer in solution. Yeast Dbr1 is also reported to be monomeric in solution (26).

Supplementary Figure S3. Topology diagram of *E. histolytica* Dbr1. Circled residues are either part of the active site or involved in binding the branchpoint adenine. The color scheme matches that used throughout the manuscript.

Supplementary Figure S4. *In vivo* debranching of the *ACT1* intron. (a) The LRL deletion mutant replaced amino acids 130-158 with a tetra-alanine linker so as to not disrupt overall protein structure. (b) *E. histolytica* Dbr1 variants remain in the soluble fraction in *S. cerevisiae*. Soluble extracts were prepared and analyzed from the indicated strains as described in the Supplemental Materials and Methods. In all cases, expression levels for point mutations and deletions were similar to active, full-length enzyme.

Supplementary Figure S5. Molecular modeling of branchpoint guanosine and cytidine in place of the branchpoint adenosine in the AK65•Dbr1 co-structure. All panels are in the same orientation. (a) Branchpoint adenosine (yellow) in the AK65•Dbr1 co-structure. (b) Model of a branchpoint guanosine (green) predicts two H-bonding interactions not present in panel (a). (c) Model of a branchpoint cytidine (blue) predicts the aromatic stacking between H16 and Y64 is weakened relative to a branchpoint adenosine. (d) A model of a branchpoint cytosine base rotated 180° relative to panel (c) suggests an unfavorable steric clash with the 5′ phosphate.

Supplementary Figure S6. Annealed omit maps with coefficients mF_o-DF_c superimposed on the α and β pockets of the refined Dbr1 structures presented in this work. All atoms with density were omitted from the phase calculation, as were atoms within a 3 Å radius. (a) 1.96 Å electron density

superimposed on the structure of the sulfate•Dbr1 complex contoured at 2.8 σ. (b) 2.0 Å electron density superimposed on the structure of the 5GMP•Dbr1 complex contoured at 2.7 σ. (c) 2.1 Å electron density superimposed on the structure of the AK65•Dbr1(C14S) complex contoured at 2.8 σ. (d) 1.95 Å electron density superimposed on the structure of the AK51•Dbr1(C14S) complex contoured at 2.6 σ in which a 3′,5′-phosphodiester linkage is clearly visible in the active site.

Supplementary Figure S7. Synthesis of RNA compounds used in this study (46). (a) Synthesis of compound AK51. (b) Synthesis of compound AK65. (c) Structures of the protecting groups used for these syntheses. (d) Mass of the final products that were synthesized.

Supplementary Figure S8. Comparison of the Dbr1 CTD to other MPE enzymes. (a) The structure of Dbr1 in complex with a model branchpoint RNA is shown for comparison (see main text **Fig. 5**). The MPE, CTD and LRL are colored as throughout the main text. (b) The CTDs of the DNA double-strand break repair enzymes Mre11 from *Thermotoga maritima* (58) and *Pyrococcus furiosus* (59) are located near the active site and are thought to serve as specificity determinants for binding substrate DNA. (c) The CTD of the 5′-nucleotidase (5′-NT) from *Escherichia coli* positions substrate nucleotides in the active site. The CTD of 5′-NT is also capable of binding substrate without the help of the MPE domain and two distinct crystal structures have shown the CTD can undergo a 96° rotation relative to the MPE domain while still retaining substrate (60). Several other MPE enzymes possess CTDs that are positioned near the active site through reciprocal "swapping" interactions. For example, (d) the cyclic AMP hydrolyzing enzyme Rv0805 from *Mycobacterium tuberculosis* is a dimer in which the C-terminal region participates in the formation of the dimer interface and in the binding of substrate in the active site of the neighboring protein molecule (22). The C-terminal region of Rv0805 also dictates localization of the enzyme to the cell wall of *M. tuberculosis* through an unresolved mechanism (22). Similar to Rv0805, the (e) C-terminal region of the hexameric glycerophosphodiesterase GpdQ from *Enterobacter aerogenes* is positioned at a domain interface and makes contacts with neighboring active sites within the hexamer (61,62).

Supplementary Table 1

	SeMet derivative				SO_4	5GMP	AK65	AK51
PDB code					4PEF	4PEG	4PEH	4PEI
**Data collection**								
Space group		$P2_12_12_1$			$P2_12_12_1$	$P2_12_12_1$	$P2_12_12_1$	$P2_12_12_1$
Cell dimensions								
a, *b*, *c* (Å)		73.4, 141.4, 214.9			73.2, 141.7, 213.2	72.8, 141.5, 212.9	73.0, 141.9, 213.7	73.0, 142.3, 213.7
	Peak	*Inflection*	*Remote*					
Wavelength (Å)	0.97918	0.97934	0.97166		1.2863	1.2823	0.9795	0.9795
Resolution (Å)	3.0 (3.05-3.00)*	2.8 (2.85-2.80)	3.2 (3.26-3.20)		1.96 (2.07-1.96)	2.00 (2.11-2.00)	2.10 (2.21-2.10)	1.95 (2.06-1.95)
R_{sym} (%)	17.1 (46.6)	12.1 (50.0)	15.3 (45.9)		7.5 (50.0)	8.3 (49.9)	8.1 (52.1)	9.7 (51.0)
$I/\sigma I$	11.8 (4.1)	7.7 (1.8)	6.2 (2.0)		13.3 (2.6)	13.0 (3.1)	11.0 (2.8)	9.1 (2.1)
Completeness (%)	99.3 (99.1)	96.3 (88.2)	98.0 (95.6)		97.2 (92.0)	96.7 (96.4)	99.5 (99.4)	99.1 (100)
Redundancy	7.5 (7.2)	2.3 (2.2)	2.3 (2.1)		4.8 (4.5)	4.9 (5.0)	4.8 (5.0)	4.0 (4.1)
**Phasing**				**Refinement**				
Resolution (Å)	2.8	2.8	2.8	Resolution (Å)	43.2-1.96	42.5-2.00	39.0-2.10	46.4-1.95
Phasing power (iso/ano)	NA/1.39	0.29/0.64	0.50/0.29	No. reflections	154,589	143,707	129,111	161,552
R_{cullis} (iso/ano)	NA/0.73	0.63/0.91	0.69/0.98	$R_{work/}$ R_{free} (%)	19.2/23.2	19.7/22.9	20.3/24.7	18.4/22.9
				No. atoms				
No. of Se sites		40		Protein	14,368	14,331	14,310	14,365
Figure of merit		0.43**		Ligand/ion	73	162	448	334
				Water	1,104	1,070	1,007	1531
				B-factors ($Å^2$)				
				Protein	26.9	32.7	40.1	24.5
				Ligand/ion	37.9	56.7	59.6	39.4
				Water	30.2	38.3	41.5	30.8
				R.m.s deviations				
				Bond lengths (Å)	0.008	0.004	0.005	0.014
				Bond angles (°)	1.135	0.902	0.907	1.498

*Highest resolution shell is shown in parentheses.
**before density modification

Supplementary Figure S1

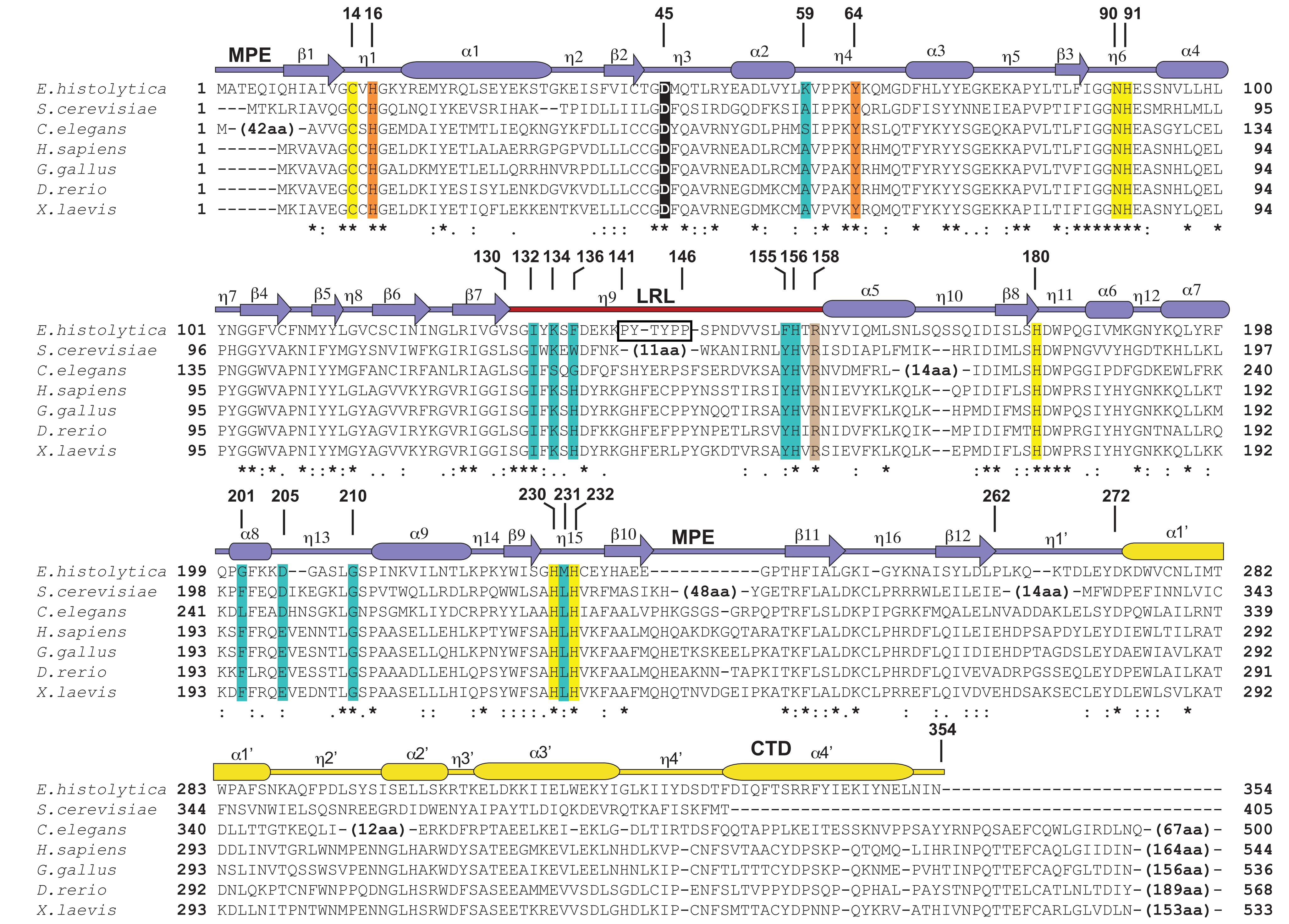

Supplementary Figure S2

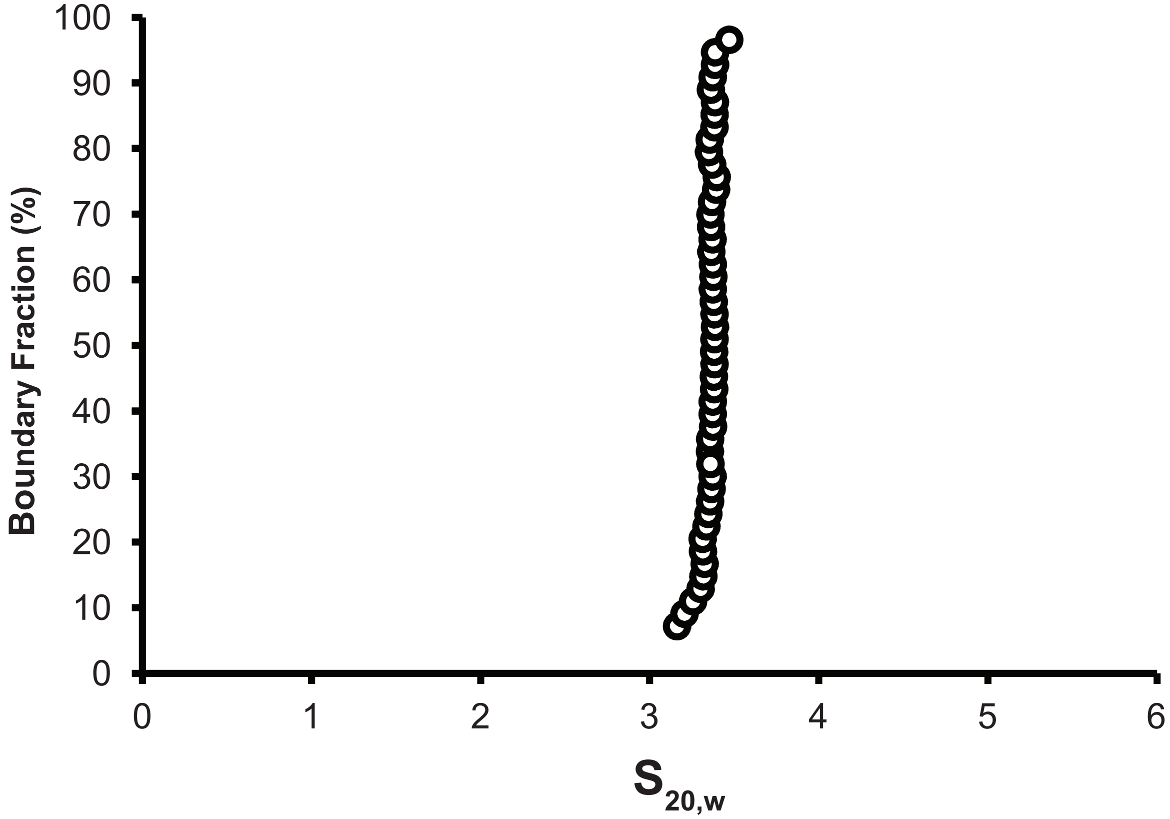

Supplementary Figure S3

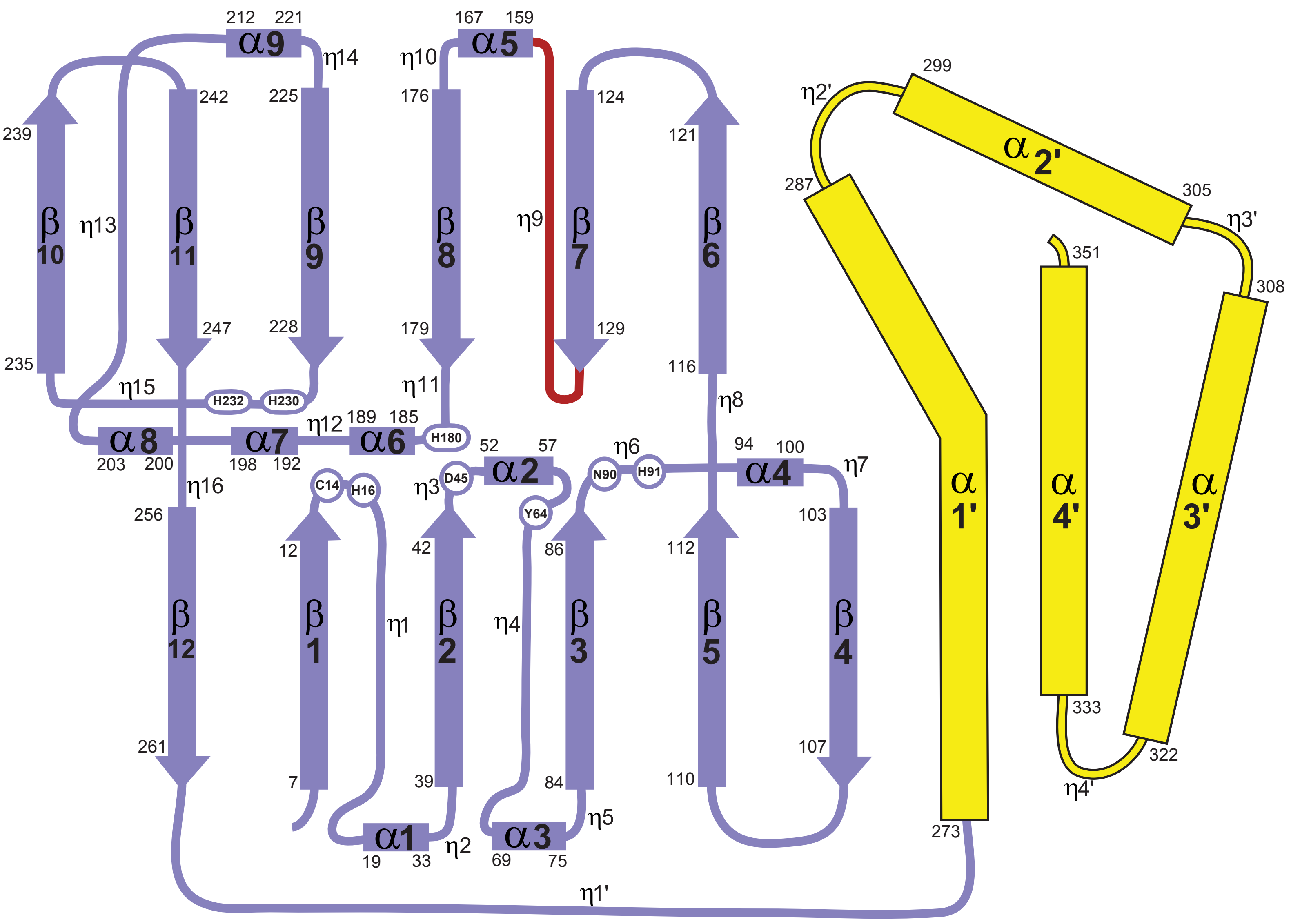

Supplementary Figure S4

(a)

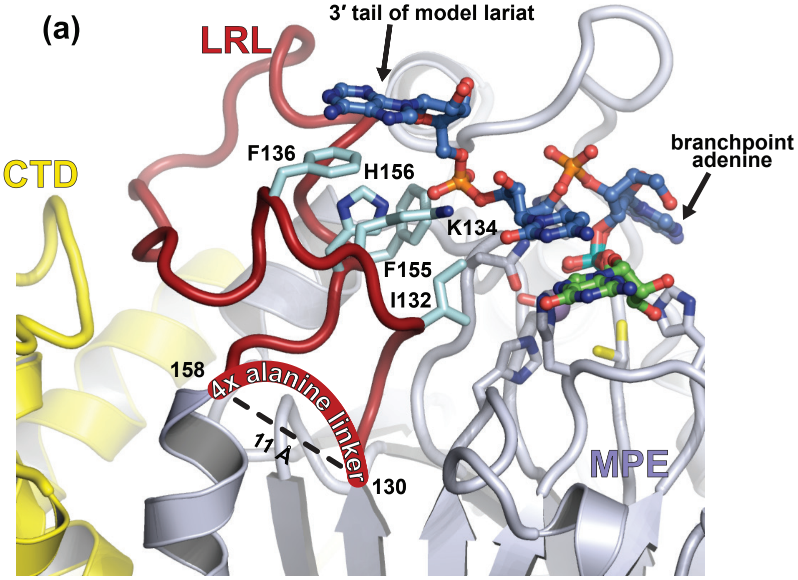

(b)

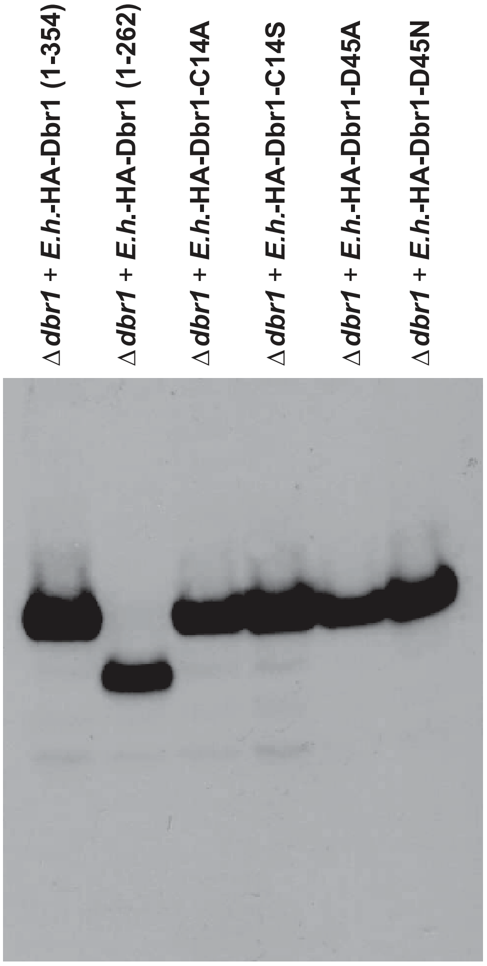

Supplementary Figure S5

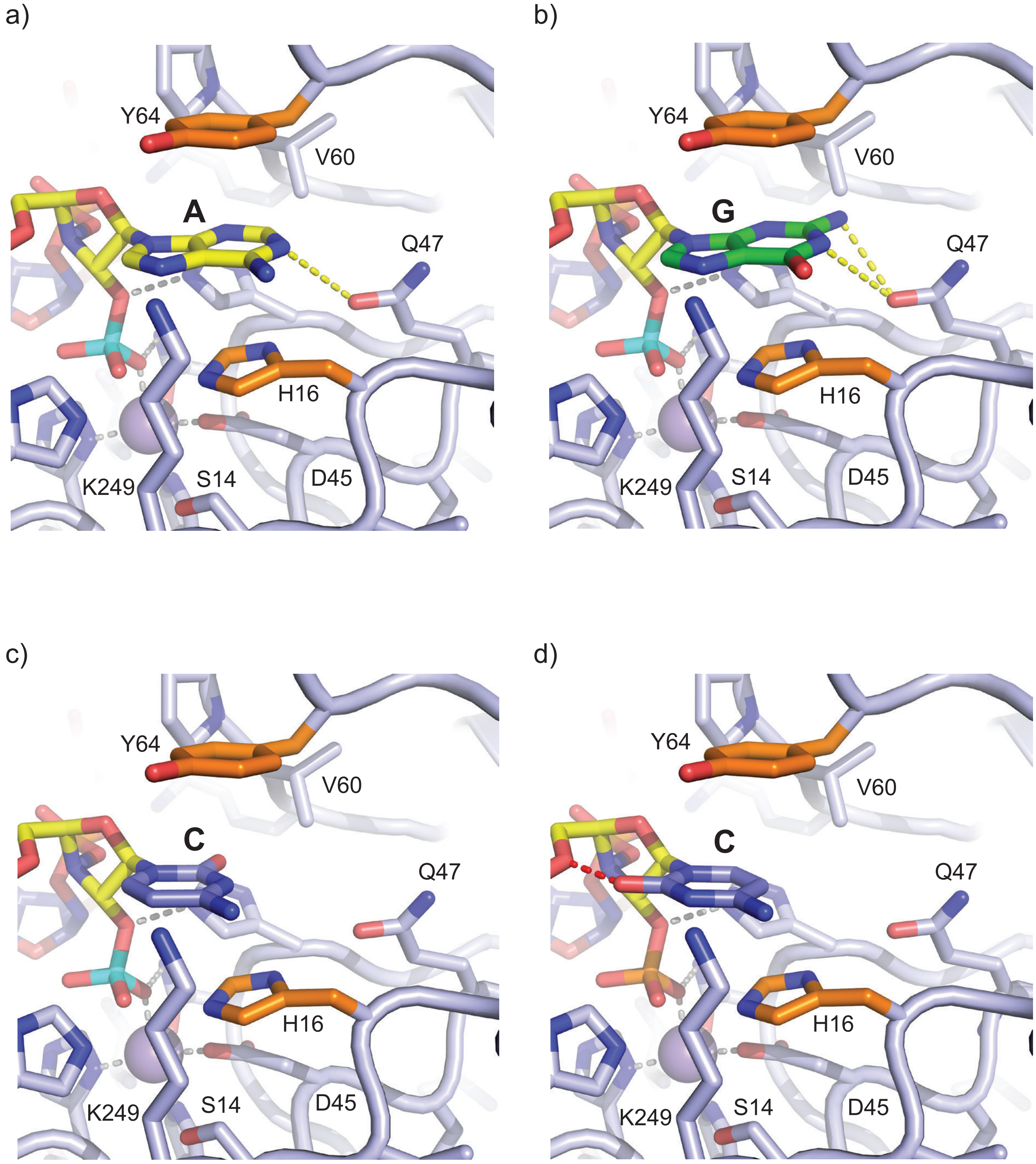

Supplementary Figure S6

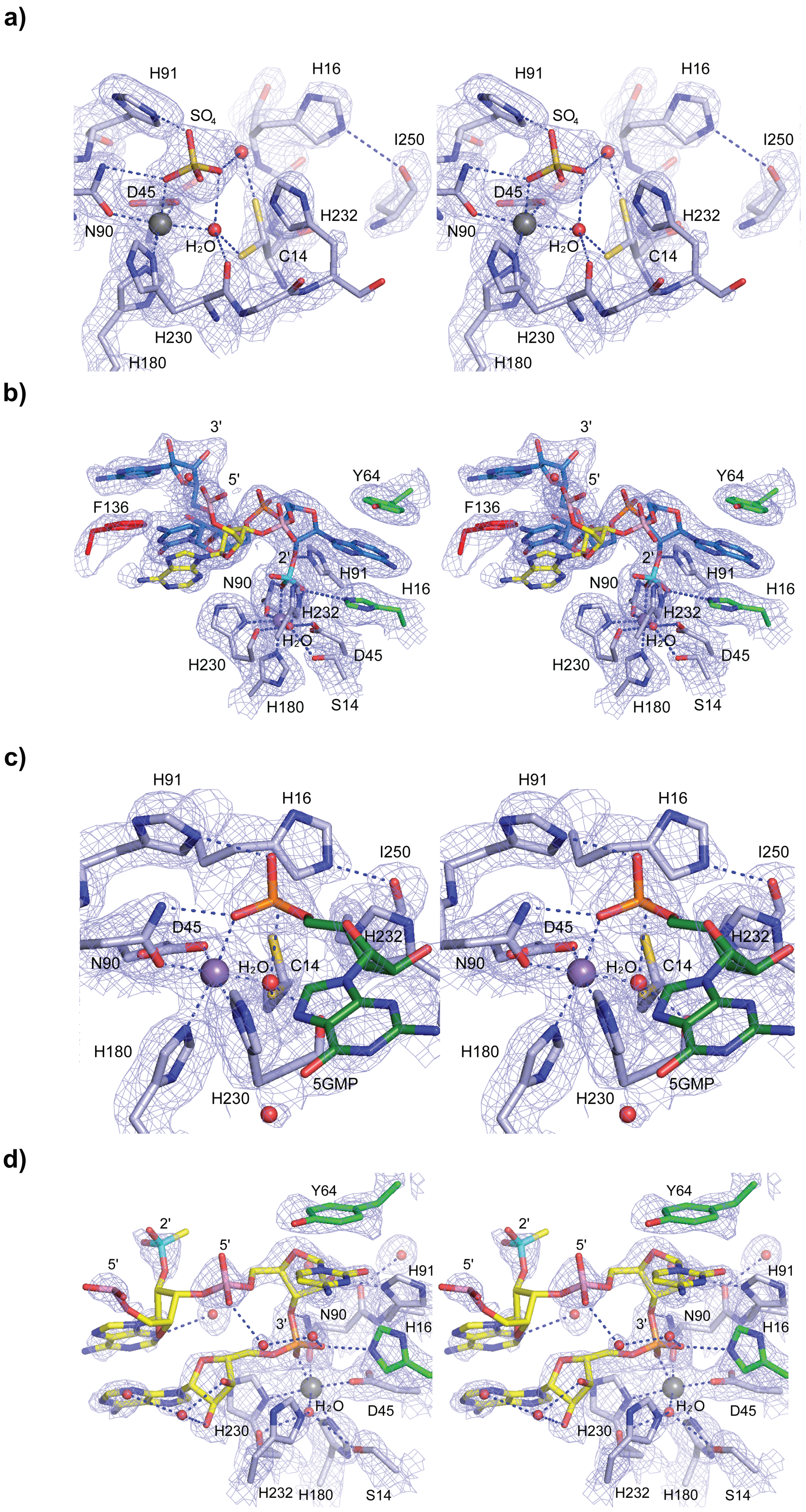

Supplementary Figure S7

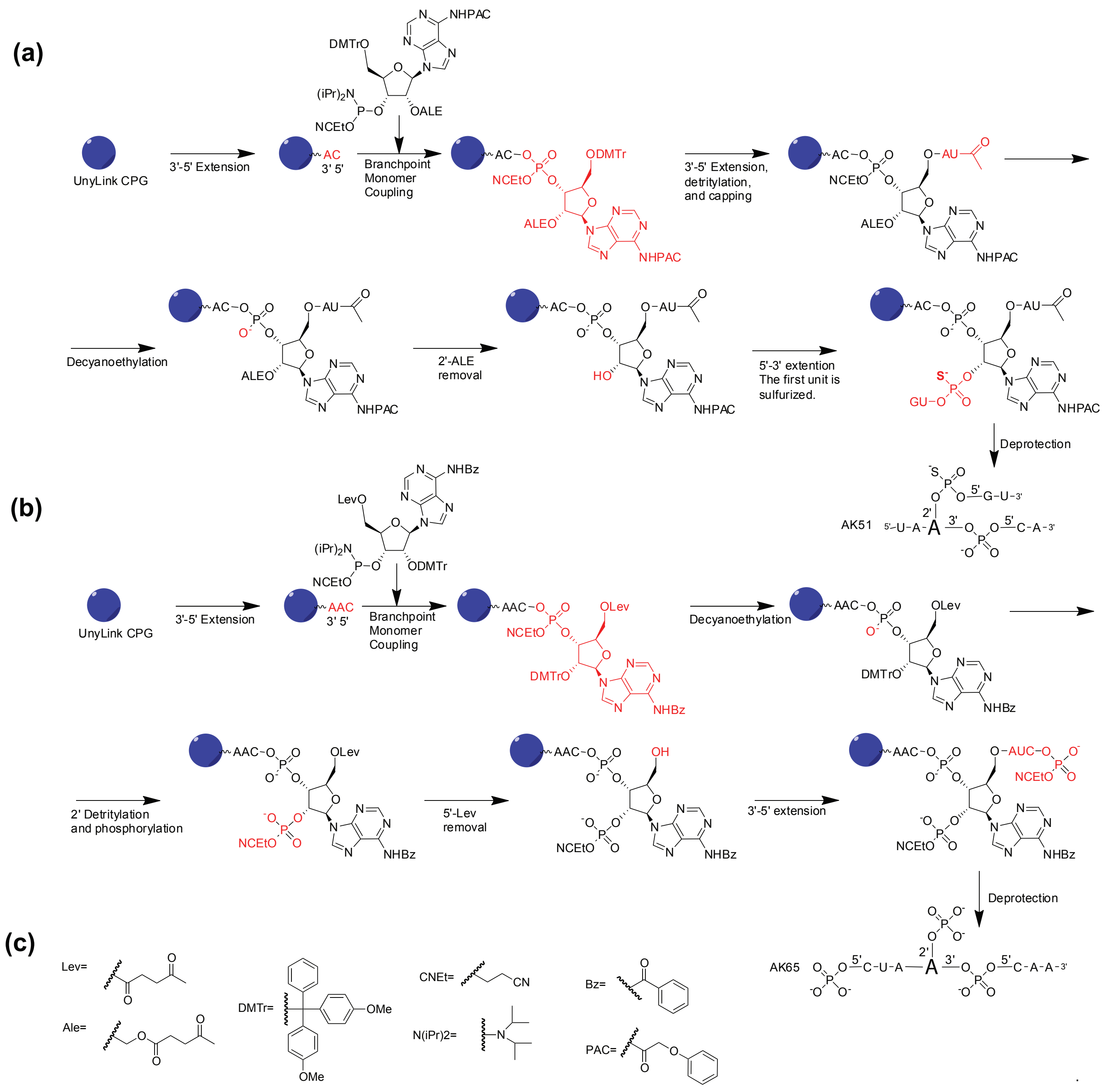

(d)

ESI-MS Characterization

Sample	Calculated Exact Mass	M/Z (doubly charged)	Mass (M/Z *2+1.007*2)
AK51	2203.318	1100.6	2203.214
AK65	2330.295	1164.0	2330.014

Supplementary Figure S8

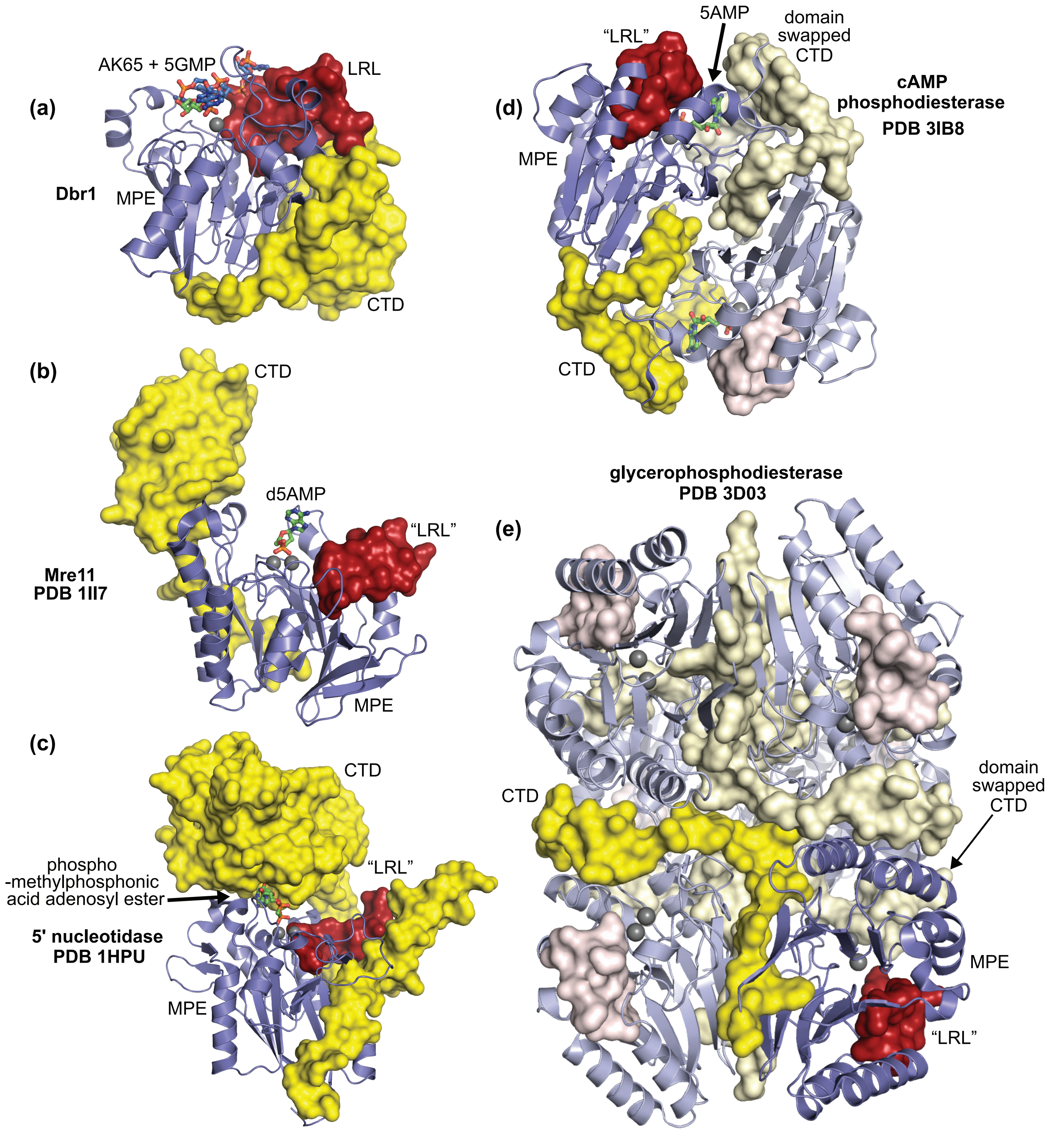

Supplement: SUPPLEMENTARY DATA [file supp_gku725_nar-01500-r-2014-File009.pdf]
